# *Clitoria ternatea*: Perspectives on Its Application in Foods and Potential Health Benefits

**DOI:** 10.3390/plants14213322

**Published:** 2025-10-30

**Authors:** Nicole Marina Almeida Maia, Irene Andressa, Jeferson Silva Cunha, Nataly de Almeida Costa, Eduardo Basílio de Oliveira, Bruno Ricardo de Castro Leite Júnior, Érica Nascif Rufino Vieira

**Affiliations:** 1 Department of Food Science and Technology, Federal University of Viçosa, Viçosa 36570-900, MG, Brazil; nicole.maia0592@gmail.com (N.M.A.M.); irene.andressa@ufv.br (I.A.); eduardo.basilio@ufv.br (E.B.d.O.); bruno.leitejr@ufv.br (B.R.d.C.L.J.); 2 Department of Food Science and Technology, Federal Institute of Education, Science and Technology of Southeast of Minas Gerais, Campus Rio Pomba, Rio Pomba 36180-000, MG, Brazil; nataly.costa@ufv.br

**Keywords:** phenolic compounds, anthocyanins, health-promoting properties

## Abstract

In recent years, edible flowers have gained increasing attention as unconventional foods, primarily due to their richness in bioactive compounds. Within this context, *Clitoria ternatea* L. (Fabaceae), commonly known as butterfly pea, stands out not only for its remarkable biological properties but also for its intense blue pigmentation. This review aims to provide a comprehensive overview of the plant’s potential in the food industry, highlighting its bioactive compounds, technological applications, and associated health benefits. Recent studies have demonstrated its antioxidant, antidiabetic, anti-obesity, hepatoprotective, and anticancer activities, as well as its use as a natural colorant, functional ingredient, active packaging component, and in nutraceutical and cosmetic formulations. Despite these promising findings, most available evidence comes from preclinical studies, with limited clinical validation to date. Therefore, further human studies are needed to confirm the efficacy and safety of the reported beneficial effects. Altogether, *C. ternatea* represents a promising natural resource for developing functional foods that meet the growing clean-label demand, fostering the incorporation of sustainable and natural ingredients.

## 1. Introduction

The use of edible flowers in human nutrition has historically been linked to cultural practices and, more recently, to the growing demand for foods that offer sensory appeal, nutritional value, and positive health effects. Edible flowers are classified as Unconventional Food Plants (UFPs) and represent a promising niche market for producers, with *C. ternatea* L. (Fabaceae) standing out due to its potential as a natural blue colorant [1,2].

In recent years, the plant-based foods market has shown robust growth, reflecting the increasing consumer demand for natural, healthy, and sustainable ingredients [3]. For example, the global plant-based foods market was valued at approximately USD 43.8 billion in 2023 and is projected to reach nearly USD 85 billion by 2030 (CAGR~9.95%) [4]. These data highlight not only the growing preference for plant-based diets but also a favorable scenario for innovations involving natural ingredients, such as plant-derived colorants.

The *C. ternatea*, commonly known as butterfly pea or “cunhã”, is a perennial herbaceous legume native to Indonesia that can reach heights of 2–3 m. It is widely distributed across equatorial tropical regions and demonstrates remarkable adaptability to a range of temperature and humidity conditions [5]. Its flowers exhibit an intense blue hue due to the presence of ternatins, attracting growing interest as a natural alternative to synthetic colorants.

Numerous studies have identified bioactive compounds in various parts of the plant, including seeds, roots, flowers, and leaves, highlighting *C. ternatea’s* potential for diverse industrial applications. In the food sector, these compounds are used as natural colorants and are also recognized for their antioxidant and antimicrobial properties. Furthermore, scientific evidence suggests potential health-promoting effects, including antidiabetic activity and hepatoprotective benefits [6].

Despite this recognized potential, previous studies and reviews have often addressed *C. ternatea* in a fragmented way, focusing mainly on phytochemistry or its role as a natural colorant. To date, there is still no integrative and updated review that consolidates the functional properties and technological applications of this field in the food sector, particularly in emerging areas such as active packaging.

In this context, the present study aims to provide a comprehensive and up-to-date overview of *C. ternatea*, highlighting its key phytochemical characteristics, functional properties, and potential applications in the food industry, with particular emphasis on its use as a natural colorant and as an active agent in packaging formulations. The study was conducted following the methodology of an integrative literature review. Although not a systematic review, the literature search and selection followed structured and transparent steps inspired by PRISMA guidelines to ensure methodological rigor and reproducibility. The search covered the last ten years of scientific production. It was carried out in the following databases: ScienceDirect (https://www.sciencedirect.com/), Scopus (https://www.scopus.com/home.uri), SciELO—Scientific Electronic Library Online (https://scielo.org/), and the CAPES/MEC Periodicals Portal (https://www.periodicos.capes.gov.br/). The search was performed using the keyword “*Clitoria ternatea*”, resulting in 1942 records. The inclusion criteria comprised original research and review articles, whereas theses, dissertations, conference papers, and book chapters were excluded. After removing duplicate records, a total of 1475 articles were retained for analysis.

## 2. Consumption, Market, and Processing of Edible Flowers

The use of flowers in human nutrition dates back to ancient times and has been associated not only with cultural practices but also with nutritional and therapeutic benefits [7]. In contemporary gastronomy, their consumption has gained popularity mainly due to the unique sensory experiences they provide, as well as their appeal as natural ingredients that can enhance the functional value of dishes, in addition to contributing flavor, color, and aroma [8]. Moreover, edible flowers are rich in bioactive compounds with antioxidant, antimicrobial, and nutraceutical properties, making them particularly attractive for the food industry [9].

The increasing consumer preference for foods and additives—such as colorants and flavorings—of natural origin is driven by the health concerns associated with artificial additives, further reinforcing this emerging market niche [8]. Despite its growth, the edible flower market still suffers from a lack of comprehensive data on production, export, and imports. Key challenges for producers include seasonality and high perishability, which limit the feasibility of exclusive cultivation [10].

To address these challenges, farmers often implement intercropping systems with cut flowers, aromatic herbs, and lettuce to enhance the value of their products [11]. Additionally, the stringent quality standards for consumption require extensive selection and discarding of flowers, which entails significant investment in production management.

Edible flowers are highly perishable due to their elevated water content, volatile compounds, and susceptibility to environmental factors that promote microbial growth [12]. During post-harvest storage, improper handling can result in tissue darkening, dehydration, petal discoloration, and abscission due to catabolic and enzymatic processes within plant cells. Therefore, fresh edible flowers must be stored under low-temperature conditions, which increases storage and transportation costs [8].

Furthermore, the absence of regulation by international authorities such as the Food and Agriculture Organization (FAO), the World Health Organization, and the U.S. Food and Drug Administration [10] hinders the standardization and consolidation of this market. On the official website of Brazil’s National Health Surveillance Agency (ANVISA), there is no legislation, guidance, or official documentation regarding edible flowers. Nevertheless, the Brazilian Agricultural Research Corporation (Embrapa) conducts research on edible flowers within the supply chain of UFPs and recognizes their production as a potential market niche [13].

## 3. Anthocyanins in *C. ternatea*

Anthocyanins are phenolic compounds belonging to the subclass of polyphenols, characterized by a carbon skeleton with a C6–C3–C6 configuration and consisting of 15 carbon atoms [14,15]. These molecules exhibit strong absorption in the UV–visible region of the electromagnetic spectrum, imparting a wide range of colors to plant tissues such as flowers, leaves, seeds, tubers, roots, stems, and fruits, with hues ranging from red to violet and blue [14,16].

The flavylium cation represents the fundamental structural form of anthocyanins. Due to its electron-deficient nature, it is highly reactive. The basic flavylium structures are typically glycosylated molecules that exhibit light absorption around 500 nm [17]. The colorimetric parameters (hue and saturation) and stability of anthocyanins are strongly influenced by the substituents attached to the anthocyanidin structure. Hydroxyl groups (–OH) tend to decrease molecular stability, whereas methoxy groups (–CH_3_) enhance it [14,18].

Modifications in the anthocyanin core structure can also affect the visible color. For example, an increase in hydroxyl substitutions is generally associated with bluish hues, while a higher number of methoxy groups tends to produce reddish tones. In anthocyanins, sugar residues may be acylated with cinnamic or aliphatic acids—organic acids naturally occurring in plant cell structures. According to the number of acyl substituents, anthocyanins can be classified as non-acylated, monoacylated, or polyacylated [19]. Polyacylation, which involves two or more acyl groups in the same molecule, often promotes intramolecular copigmentation between the aromatic acyl moieties and anthocyanidins. This phenomenon is responsible for the characteristic bluish coloration of certain flowers, even under physiological pH conditions, as observed in *C. ternatea* [1].

Polyacylated anthocyanins display superior coloring potential due to their enhanced stability across a wide pH range [20]. *C. ternatea* accumulates a series of polyacylated anthocyanins in its petals, known as ternatins (A1, A2, A3, B1, B2, B3, B4, C1, C2, C3, C4, C5, D1, D2, and D3) [1,21]. Anthocyanins are water-soluble pigments primarily stored in vacuoles. Most are derived from pelargonidin, cyanidin, or delphinidin, with the degree of hydroxylation on the B ring determining the color range from red to blue [22].

A review study reported that the extraction of anthocyanins is traditionally mediated by solvents, such as water, ethanol, methanol, and acetone, either individually or in combination. However, it is emphasized that the use of green and sustainable solvents has become increasingly relevant. Regarding extraction techniques, in addition to the conventional solvent-based method, emerging strategies, such as ultrasound-assisted extraction, microwave-assisted extraction, supercritical fluid extraction, and pressurized liquid extraction, have been employed to enhance efficiency, selectivity, and the preservation of the integrity of these bioactive molecules [23].

In human metabolism, anthocyanins play multiple biological roles, with antioxidant activity being the most notable. In addition, their potential use as natural colorants represents a valuable technological feature for the food industry [24].

## 4. *C. ternatea*: Functional Properties, Health Benefits, and Applications in the Food Industry

*C. ternatea* is native to the Maluku Archipelago (Indonesia), with its first documented occurrence on the island of Ternate. Today, it is widely distributed across South and Central America, tropical regions of Asia such as India, China, and the Philippines, and other equatorial tropical countries like Brazil. Several cultivars of *C. ternatea* exhibit a range of flower colors, including dark blue, light blue, lilac, and white. This color diversity is primarily attributed to the chemical structures of the various anthocyanins present in the petals [22].

Traditionally, *C. ternatea* has been employed in classical Indian Ayurvedic medicine for the treatment of various ailments, and scientific studies have corroborated the wide-ranging pharmacological activities of its bioactive compounds [25]. These compounds are not only linked to health-promoting effects but also hold potential for use as functional ingredients and additives in the food industry. The following sections provide a detailed overview of its principal functional properties, health benefits, and applications in food production.

### 4.1. Functional and Health-Related Claims

Phenolic compounds are the most abundant bioactive constituents in *C. ternatea*, particularly ternatins and delphinidin derivatives, which are responsible for the intense blue coloration of the petals [6]. Chemically, phenolic compounds are characterized by aromatic rings bearing hydroxyl groups [26]. These molecules are products of plant secondary metabolism, serving not only as a defense mechanism against external stressors such as light, temperature, insects, and humidity, but also contributing to internal processes, including genetic differentiation, nutrient assimilation, and hormone synthesis [27].

In humans, phenolic compounds are associated with a range of beneficial physiological effects, including reduced risk of cardiometabolic diseases and protection against oxidative damage to lipids and lipoproteins. Their mechanisms of action include inhibition of platelet aggregation, free radical scavenging, and metal chelation [28,29].

Bioactive compounds extracted from the seeds, roots, flowers, and leaves of *C. ternatea* have been extensively studied for their antidiabetic, anticarcinogenic, hepatoprotective, anti-obesity, antioxidant, and hepatoprotective activities as summarized in Table 1.

#### 4.1.1. Antidiabetic Effect

Conventional pharmacological treatments for diabetes mellitus (DM) are effective but can cause adverse effects such as weight gain, hypoglycemia, and gastrointestinal disturbances [32]. In this regard, natural compounds, such as those present in *C. ternatea*, represent promising complementary alternatives, offering therapeutic benefits with fewer side effects compared to synthetic drugs.

The antidiabetic potential of *C. ternatea* is mainly attributed to its antioxidant activity, conferred by anthocyanins and flavonoids. These compounds protect pancreatic β-cells against oxidative stress, supporting the management of type 1 DM. In addition, they interact with α-amylase, a key digestive enzyme responsible for carbohydrate hydrolysis. Inhibition of this enzyme delays glucose release in the intestine, thereby reducing postprandial blood glucose levels [45].

Furthermore, bioactive compounds such as flavonoids, anthocyanins, and alkaloids improve insulin production and enhance cellular sensitivity to the hormone, suggesting that *C. ternatea* could be applied not only in diabetes management but also in mitigating its associated complications, including cardiovascular diseases and cognitive impairment [41]. These beneficial effects of *C. ternatea* have been demonstrated in both *in vivo* and *in vitro* studies, as summarized in Table 1. Importantly, recent human clinical studies have begun to support these findings. In a randomized crossover trial with 15 healthy male volunteers, acute consumption of *C. ternatea* flower extract significantly suppressed postprandial plasma glucose and insulin levels when co-administered with sucrose, while enhancing antioxidant capacity without inducing fasting hypoglycemia [41]. Additionally, a 12-week study in patients with type 2 diabetes experiencing moderate diabetic distress showed that daily administration of the extract (5–10 g) significantly reduced serum protein carbonyls and increased urinary 5-HIAA levels, indicating improved oxidative stress status and potential benefit in diabetic distress management [46].

These findings highlight that, besides preclinical evidence, clinical studies in humans support the antidiabetic and antioxidant potential of *C. ternatea*. However, further trials with larger populations are needed to confirm these effects and establish optimal doses for therapeutic use.

#### 4.1.2. Anticarcinogenic Activity

Extracts of *C. ternatea* have been extensively investigated due to their intrinsic biological properties, as shown in Table 1. Among these, the potential anticancer activity stands out, which has attracted growing scientific interest regarding the incorporation of this plant into pharmaceutical formulations for the treatment of different cancer types [47,48]. This potential is mainly attributed to the high levels of flavonoids and phenolic compounds present in *C. ternatea* extracts, which act through cellular mechanisms such as apoptosis induction, inhibition of cell proliferation, and modulation of signaling pathways involved in tumor development [33,49].

Shen et al. [50] evaluated the anticancer effect of hydrophilic (methanol) and lipophilic (ethyl acetate and hexane) extracts from petals and seeds of *C. ternatea* on the viability of laryngeal carcinoma cells. The study used a human HEp-2 carcinoma cell line and identified a wide range of phenolic compounds, flavonoids, and ternatins in the extracts. Hydrophilic extracts from seeds and petals reduced HEp-2 cell viability by 95% at a concentration of 1 mg/mL. By contrast, lipophilic extracts only showed cytotoxic effects at higher concentrations (6.0–9.0 mg/mL) and were less effective than hydrophilic extracts. The authors attributed this difference to the higher concentration and distinct profile of phenolic compounds in hydrophilic extracts, which block key enzymes and glucose transporters, induce apoptosis, and thereby directly reduce HEp-2 cell viability.

In another study, HER2-positive breast cancer MCF-7 cells treated with crude flower extract of *C. ternatea* at concentrations below the IC50 (estimated at 862 µg/mL) showed a 50% reduction in migratory capacity. These findings suggest that the extract has the potential to inhibit the metastatic activity of this cell line *in vitro* [51]. Altogether, these results underscore the promising role of *C. ternatea* as an anticancer agent, although further studies are required to validate its therapeutic efficacy in more complex biological models.

#### 4.1.3. Hepatoprotective Activity

The hepatoprotective activity of *C. ternatea* extract has been previously described [36] (Table 1). In an experimental model of diabetes and dyslipidemia in rats, treatment with *C. ternatea* significantly reduced aspartate aminotransferase (AST) and alanine aminotransferase (ALT) levels, two key biomarkers of liver injury. Moreover, notable improvements were observed in the histological integrity of the liver, including decreased tissue inflammation and restoration of hepatic architecture. Steatosis, inflammatory infiltration, and necrosis were markedly attenuated when compared to the control group [52].

Similarly, in a model of paracetamol-induced hepatotoxicity, administration of a methanolic flower extract of *C. ternatea* (200 mg/kg) in mice significantly reduced serum AST, ALT, and bilirubin levels compared with the toxic control group (*p* < 0.01). Histological analysis further confirmed protective effects on liver tissue, with improvements in cellular organization and architecture.

Collectively, these findings demonstrate that *C. ternatea* exerts consistent hepatoprotective effects across different models of liver injury, whether of metabolic or toxic origin. Reduced serum biomarkers of hepatocellular damage and preservation of histological structure evidence its efficacy. These protective effects are primarily attributed to its rich composition of bioactive compounds, particularly anthocyanins, with antioxidant and anti-inflammatory properties, which neutralize oxidative stress and modulate cellular pathways involved in inflammation and apoptosis [53]. Thus, *C. ternatea* extract represents a promising natural therapeutic candidate for the prevention and mitigation of liver damage.

#### 4.1.4. Anti-Obesity

Recent studies have highlighted the potential antidiabetic effects of *C. ternatea*, attributed to its ability to inhibit digestive enzymes such as α-amylase and α-glucosidase, which are directly involved in carbohydrate breakdown and glucose absorption in the intestine [38,54].

Furthermore, clinical trials have demonstrated that *C. ternatea* flower extract can modulate postprandial metabolic responses in overweight and obese individuals. For instance, Thilavech et al. [38] reported that ingestion of 2 g of the extract with a high-fat meal significantly attenuated postprandial lipemia, reducing the area under the triglyceride curve and free fatty acid levels up to 360 min after the meal. In addition, plasma antioxidant capacity was significantly enhanced, as evidenced by increased sulfhydryl group levels and higher glutathione peroxidase activity, indicating improved defenses against diet-induced oxidative stress. These effects suggest that *C. ternatea* consumption may contribute to lipid metabolism regulation and oxidative stress reduction. A summary of these anti-obesity effects is presented in Table 1.

*In vitro* studies with 3T3-L1 adipocytes further support the anti-obesity potential of *C. ternatea*. Chayaratanasin et al. [55] demonstrated that the extract inhibited adipogenesis at multiple stages, initially suppressing preadipocyte proliferation and cell cycle progression by downregulating Akt and ERK1/2 signaling pathways. During differentiation, it reduced the expression of adipogenic transcription factors PPARγ and C/EBPα, as well as lipogenic enzymes fatty acid synthase (FAS) and acetyl-CoA carboxylase (ACC), leading to decreased intracellular triglyceride accumulation. Moreover, the extract enhanced catecholamine-induced lipolysis, promoting the mobilization of stored fat.

Additionally, *C. ternatea* flower extracts exhibit significant antioxidant activity, which may reduce oxidative stress associated with insulin resistance and the progression of type 2 diabetes [56]. *In vivo* evidence also suggests that supplementation with these extracts can lower fasting blood glucose, improve insulin sensitivity, and positively modulate gene expression related to glucose metabolism.

Sasmana et al. [57] reported that aqueous extracts of *C. ternatea* petals (CTE), rich in anthocyanins (78.09 mg/100 g) and tannins (1424.90 mg/100 g), significantly prevented high-fat-diet-induced obesity and dyslipidemia in animal models. CTE administration reduced body weight, improved lipid profile parameters (total cholesterol, LDL, VLDL), and decreased thoracic aorta tunica thickness compared with control animals. These effects were statistically significant (*p* < 0.05), highlighting CTE’s potential as a natural agent with anti-obesity, cholesterol-lowering, and vasoprotective properties.

Overall, *C. ternatea* flower extract represents a promising natural therapeutic option for obesity management. In addition to promoting weight reduction, *C. ternatea* may prevent dyslipidemia-related complications by improving HDL, LDL, and total cholesterol levels, while modulating digestive enzymes such as amylase and lipase toward optimal values. These extracts can also inhibit weight gain, enhance adipose lipolysis, and decrease the expression of adipogenic and lipogenic proteins [58].

Taken together, these findings underscore *C. ternatea* as a promising candidate for the development of functional foods and beverages with antidiabetic and anti-obesity properties, particularly for the prevention and management of metabolic disorders [59]. All these effects are summarized in Table 1, reinforcing its potential application in human nutrition.

#### 4.1.5. Antioxidant Activity

*C. ternatea* has been extensively investigated for its antioxidant properties, primarily attributed to anthocyanins, a subclass of phenolic compounds including ternatins. These anthocyanin compounds can neutralize reactive oxygen species (ROS), protect biomolecules from oxidative damage, and modulate endogenous antioxidant enzymes, playing a crucial role in preventing cellular damage associated with oxidative stress.

Recent studies have demonstrated the broad antioxidant potential of this plant across various matrices and experimental models. Padmanabhan and Parvatam [60] examined both the oil extracted from *C. ternatea* seeds and the defatted seed cake produced during extraction. Both products displayed significant antioxidant capacity and *in vitro* anti-inflammatory effects, highlighting the potential for comprehensive utilization of the plant as a source of anthocyanins.

Similarly, Prasad et al. [61] evaluated the effects of *C. ternatea* extract on human glioblastoma cells, an *in vitro* model representative of the nervous system. Treatment with the extract significantly decreased intracellular ROS levels, enhanced mitochondrial membrane potential—a marker of cellular integrity—and activated DNA repair and cell differentiation pathways. These findings indicate that the plant’s bioactive compounds can protect sensitive tissues, such as neural cells, against oxidative stress.

Other studies underscore the complexity and breadth of *C. ternatea* antioxidant effects. Partially purified flower extracts exhibited antioxidant activity against ROS, antimicrobial effects, protection against erythrocyte hemolysis, inhibition of digestive enzymes (α-amylase and α-glucosidase) and angiotensin-converting enzyme, suppression of lipid peroxidation, free radical scavenging, DNA strand protection, and inhibition of LDL cholesterol oxidation *in vitro* [28]. These effects are primarily attributed to the anthocyanins, whose positively charged structures enable self-association and interactions with other flavonoids, enhancing their antioxidant potential [28].

Analysis of blue flowers by Bragueto et al. [62] demonstrated that aqueous petal extracts exhibited *in vitro* antioxidant activity, protective effects against human erythrocyte hemolysis, and potential antihypertensive and antidiabetic effects. In this context, Widowati et al. [35] highlighted the multifunctional potential of *C. ternatea* flowers as antioxidant, anti-inflammatory, and antidiabetic agents, due to flavonoids such as delphinidin, rutin, kaempferol, malvidin, and quercetin, and the complex chemical composition of the extracts, including not only anthocyanins, but also other bioactive phenolics, which together enhance the overall antioxidant potential. According to Escher et al. [28], these compounds are well-recognized for their radical-scavenging activity and are central to the protective effects observed under various pathophysiological conditions.

Taken together, these findings indicate that *C. ternatea* is a promising source of natural antioxidants with potential applications in functional foods, phytotherapeutics, and nutraceutical products aimed at preventing and managing chronic diseases associated with oxidative stress, including diabetes, hypertension, and neurodegenerative disorders. These effects are summarized in Table 1.

#### 4.1.6. Anti-Inflammatory Activity

*C. ternatea* extracts have demonstrated remarkable potential as anti-inflammatory agents (Table 1), as evidenced by a range of experimental approaches exploring their bioactive constituents and underlying mechanisms of action.

Wang et al. [37] investigated the effects of a blue petal extract of *C. ternatea* on inflammation induced in C57BL/6 mice. The extract was found to be rich in flavonoids, with nine compounds tentatively identified. Male C57BL/6J mice were fed either a standard diet (SD) or a high-fat, high-fructose diet (HFFD) for 16 weeks, with the HFFD groups receiving 0.25%, 0.5%, or 2% (*w*/*w*) of the aqueous extract in their drinking water. Treatment significantly improved oxidative stress parameters and inflammatory mediators, indicating that the anthocyanins present in the blue petals exerted substantial anti-inflammatory effects while promoting reverse cholesterol transport.

Supporting these findings, Adhikary, Sultana, and Bishayi [63] reported pronounced anti-inflammatory and antiarthritic effects of *C. ternatea* petal extract (CTE) and its main bioactive compound, quercetin-3β-D-glucoside (QG), in mice with collagen-induced arthritis. Treatment with CTE (50 mg/kg) and QG (2.5 mg/kg) markedly reduced inflammatory cell infiltration, myeloperoxidase activity, and the release of pro-inflammatory cytokines and chemokines. Moreover, it decreased the production of reactive oxygen and nitrogen species (ROS/RNS) and downregulated the expression of key inflammatory proteins such as TNFR1, TLR2, COX-2, iNOS, and MMP-2 in synovial tissue. These results confirm the extract’s ability to modulate pro-inflammatory signaling pathways and mitigate oxidative stress, reinforcing its therapeutic potential in managing chronic inflammatory diseases such as arthritis.

Expanding on the anti-inflammatory potential of the species, Padmanabhan and Parvatam [60] evaluated oils extracted from the mature seeds of blue-flowered (BSO) and white-flowered (WSO) varieties of *C. ternatea*. Both oils exhibited high levels of total phenolics (55–63 mg/100 g) and flavonoids (18–24 mg/100 g), including quercetin (67–116 mg/100 g) and sinapic acid (0.08–0.31 mg/100 g), which contributed to their notable antioxidant capacity. An *in vitro* protein denaturation inhibition assay revealed significant anti-inflammatory activity, suggesting that these phenolic and flavonoid compounds modulate inflammatory processes through protein stabilization and attenuation of oxidative stress. These findings position *C. ternatea* seed oils as promising sources of bioactive compounds for use in functional foods and nutraceutical formulations targeting inflammation control.

More recently, Permatasari et al. [64] demonstrated the strong anti-inflammatory potential of kombucha produced from *C. ternatea* flowers. In mice fed a high-fat, high-cholesterol diet, consumption of the fermented beverage significantly reduced the levels of pro-inflammatory cytokines TNF-α and PGC-1α, while enhancing the expression of the anti-inflammatory cytokine IL-10, thereby restoring systemic inflammatory balance. Improvements in oxidative stress and beneficial modulation of gut microbiota—both closely associated with inflammatory regulation—were also observed. These results further support that *C. ternatea*-derived products, such as kombucha, can effectively prevent or attenuate low-grade chronic inflammation linked to lipid-rich diets and metabolic disorders.

Taken together, these studies provide compelling evidence that *C. ternatea* is a promising natural source of bioactive compounds with multifaceted anti-inflammatory mechanisms, including cytokine modulation, oxidative stress reduction, and tissue protection. Collectively, the findings underscore its potential for application in complementary therapies, functional foods, and nutraceutical formulations aimed at promoting health and mitigating chronic inflammation.

To provide an integrated overview of the evidence, Table 2 summarizes the scientific studies addressing the anticancer, anti-obesity, and anti-inflammatory effects of *C. ternatea*, highlighting the study type (*in vitro* or *in vivo*), the principal bioactive compounds involved, and molecular pathways.

Although studies demonstrate the functional properties of *C. ternatea* anthocyanins, bridging the gap between experimental findings and their effective therapeutic or industrial application remains challenging. Yu et al. [68] investigated the bioavailability of anthocyanins from *C. ternatea* extracts in rats and reported that only a small fraction is absorbed. In addition, there is currently no standardized method for preparing *C. ternatea* extracts, and different studies use varying extraction protocols, as shown in Table 1. This lack of standardization limits reproducibility and the reliability of functional outcomes. These factors raise concerns about efficacy, formulation stability, and regulatory compliance. Therefore, rigorous clinical trials and comprehensive regulatory evaluation are required to ensure both the safety and the functional consistency necessary for the broader industrial and pharmaceutical use of *C. ternatea* [69].

### 4.2. Technological Applications of C. ternatea

*C. ternatea* has been traditionally employed in Malaysian cuisine, such as in Nasi Kerabu, where its flowers are used to color rice blue [70] naturally. Beyond its culinary applications, the plant has also been recognized for enhancing the nutritional profile of foods [71].

In the food industry, achieving an appealing and stable color is a key aspect of product formulation, often requiring the use of color additives. Although synthetic colorants typically exhibit greater stability than natural pigments, their safety is increasingly questioned [71]. Moreover, there is a growing global demand for natural products with health-promoting properties, which drives the search for safe, stable, and functional natural colorants [72].

**Figure 1:**
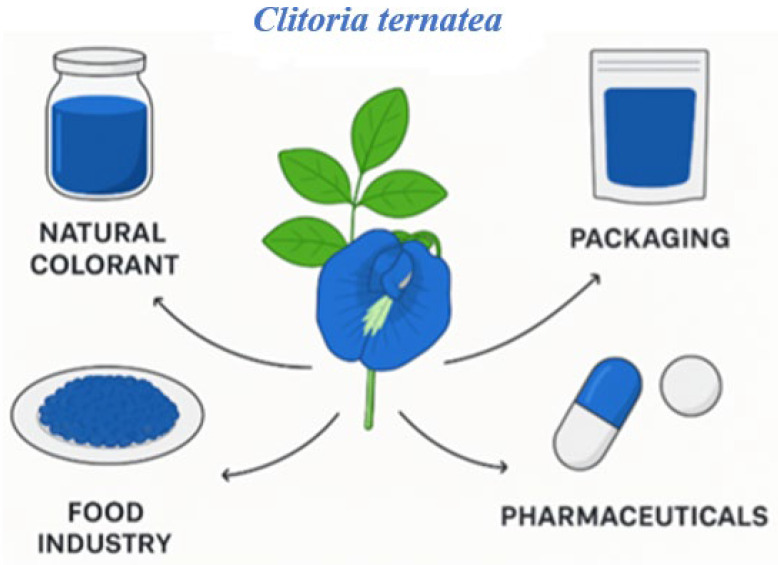
Technological applications of *C. ternatea*.

Considering these factors, *C. ternatea* can serve as a source of natural blue pigments for processed foods and has potential applications across the food, pharmaceutical, and broader industrial sectors (Figure 1), as discussed in the following sections.

#### 4.2.1. Natural Colorant

*C. ternatea* flowers are extensively utilized due to their diverse anthocyanins, which are responsible for the plant’s characteristic intense blue coloration. Among these pigments, ternatins are particularly notable—a group of approximately 15 polyacylated anthocyanins exhibiting high stability across varying pH conditions, making them highly suitable for technological applications as natural colorants. Structurally, the stability of ternatins is attributed to intramolecular copigmentation, mediated by molecular stacking between aromatic acyl residues and the anthocyanidin chromophore, with hydrophobic interactions occurring on both sides of the molecule [73].

Organic solvents such as methanol and ethanol remain the most common methods for anthocyanin extraction. However, these solvents have notable drawbacks, including volatility, flammability, toxicity, and negative environmental impacts, which has led to a gradual discouragement of their use in the pharmaceutical, cosmetic, and food industries [74,75,76].

As a sustainable alternative, natural deep eutectic solvents (NADES) have been developed, offering alignment with Green Chemistry principles [77,78]. Beyond minimizing environmental impact, NADES enable the development of functional foods and nutraceuticals incorporating natural colorants and preservatives [74]. Their advantages include tunable viscosity, biodegradability, the capacity to extract both polar and nonpolar compounds, ease of separation, and the potential for designing customized extraction systems [76].

Within this framework, ultrasound-assisted extraction (UAE) has emerged as a highly effective strategy to optimize extraction processes, reduce costs, and increase the yield of bioactive compounds. UAE has been successfully applied for obtaining natural additives in the food industry, supporting the production of clean-label products, reducing solvent consumption, and enhancing process efficiency when optimal extraction parameters are applied [79].

Moreover, the aqueous extract of *C. ternatea* exhibits pH-dependent color shifts to red, pink, or green, expanding its range of technological applications. Consequently, its use as a natural colorant represents a viable and sustainable alternative to synthetic dyes in food and beverage formulations, underscoring the potential of butterfly pea anthocyanins as functional ingredients in the food industry.

#### 4.2.2. Packaging

The incorporation of *C. ternatea* extracts has attracted increasing interest in the development of active and intelligent packaging, mainly due to the presence of acylated anthocyanins, known as ternatins. These pigments exhibit an intense blue color at neutral pH, with hues shifting across a broader chromatic range than most plant-derived anthocyanins as pH varies [80]. Such a property is auspicious for the design of freshness-indicating films for meat, fish, and other protein-rich foods, since microbial and biochemical spoilage leads to the release of volatile amines, which raise the surface pH [81].

A representative example is provided by Narayanan et al. [82], who developed films composed of pectin, eggshell membrane gelatin, and glycerol, enriched with *C. ternatea* anthocyanins to monitor the spoilage of fresh tilapia. The anthocyanin extract contained 198.3 mg/g of pigments. The films exhibited high pH sensitivity, showing red, purple, blue, green, and yellow hues across the pH range of 1–13. During refrigerated storage at 4 °C for 7 days, a visible color transition occurred, from dark blue and bluish-gray to olive-green and dark green. This colorimetric response was directly correlated with pH changes and increasing levels of total volatile elemental nitrogen (TVB-N).

In another study on seafood, Kaewprachu et al. [83] designed intelligent carboxymethylcellulose-based films incorporated with anthocyanins extracted from Karanda fruit (*Carissa carandas*) pomace and *C. ternatea* flowers. These films were applied to monitor shrimp spoilage under different storage conditions (4 °C for up to 8 days and 25 °C for up to 30 h). Distinct visual color changes allowed the discrimination of freshness stages: dark purple (fresh), purplish-gray/gray (semi-fresh), and olive-green or brown (spoiled). A positive correlation was observed between total color difference (ΔE) and spoilage indicators, especially TVB-N and total viable count (TVC). Importantly, films containing both anthocyanin sources demonstrated enhanced stability and clearer chromatic responses compared to those prepared with single extracts, highlighting the synergistic effect of pigment combinations.

In the poultry sector, Ahmad, Lim, and Navaranjan [84] developed a colorimetric indicator by immobilizing *C. ternatea* anthocyanins into a sago (*Metroxylon sagu*) matrix. The film, produced via solvent casting, was characterized by pH-dependent color change, water solubility, swelling behavior, and morphology. When applied to chicken breast samples under different storage conditions, the film displayed distinct color responses associated with pH variation during spoilage. This performance enabled real-time freshness monitoring, with precise visual detection and low production costs.

Anthocyanins from butterfly pea flowers have also been employed in colorimetric packaging for pork preservation [85]. An intelligent starch-based biopolymeric film was fabricated by incorporating carbon dots and *C. ternatea* anthocyanins. The extract not only imparted pH sensitivity but also increased the film’s water responsiveness. The films exhibited marked color variations at different pH levels, enabling their use as low-cost freshness indicators for packaged pork. During storage, a progressive color shift from purple to green was observed, in line with storage duration and spoilage progression.

In addition to their colorimetric potential, *C. ternatea* extracts display antimicrobial activity against *Escherichia coli*, *Staphylococcus aureus*, and *Pseudomonas* spp. [86]. This antimicrobial capacity is particularly advantageous for developing active or preservative packaging, as the extract can act as a natural coadjutant, providing an additional protective barrier against microbial proliferation in packaged foods.

Despite promising results regarding pH sensitivity and antimicrobial activity, the industrial application of active and intelligent films based on *C. ternatea* requires careful consideration. Key factors include production cost, process scalability, and food safety. Preliminary studies suggest that *C. ternatea* extracts can be incorporated using simple methods, such as solvent casting, and at relatively low cost, particularly when combined with abundant, inexpensive polymer matrices. Nevertheless, further studies are needed to confirm long-term stability, consistent color performance, and compatibility with various food types at an industrial scale, ensuring these technologies are practical, safe, and economically viable for commercial use.

#### 4.2.3. Other Applications

The *C. ternatea* have garnered increasing interest across various industrial and technological sectors. In the bakery sector, *C. ternatea* extracts have been shown to significantly reduce starch hydrolysis, predicted glycemic index, and overall starch digestibility by inhibiting carbohydrate-digesting enzymes in *in vitro* assays [87]. Additionally, anthocyanin-rich extracts demonstrated strong anti-biofilm activity against cariogenic bacteria and *Pseudomonas aeruginosa* [65].

Another innovative application is the use of anthocyanins as visual biosensors. Due to their pH sensitivity, these pigments have been incorporated into polymer matrices to create sensors that detect food freshness or spoilage, as well as environmental changes. This represents an advance in innovative materials for food quality and safety monitoring, with potential integration into active and intelligent packaging systems [76,88].

According to the study by Szymański, Pawlik, and Dobrucka [89], the incorporation of the floral extract into cellulose and gelatin-based films resulted in packaging with improved barrier properties and significant antioxidant activity, reducing the risk of migration of undesirable chemical compounds into food. Furthermore, the natural blue color derived from ternatein-type anthocyanins adds aesthetic and functional value. It can act as a visual indicator of environmental changes and as a marketing differentiator, highlighting the potential of *C. ternatea* in the formulation of sustainable, intelligent, and multifunctional packaging systems. Furthermore, the use of *C. ternatea* has been explored in various experimental and commercial products, including prebiotic ice cream [90], yogurt [91], drink (tea) [92] and gin [93].

Within the pharmaceutical and nutraceutical industries, petal extracts have been investigated as functional ingredients with potential applications in managing obesity, type 2 diabetes, neurodegenerative disorders, and cardiovascular diseases. Evidence suggests that these extracts can modulate insulin resistance, lower serum glucose and lipid levels, and provide neuroprotective effects, including benefits to memory and the cholinergic system [35].

Other applications of *C. ternatea* are found in the cosmetic and textile sectors, highlighting the versatility of this plant. In the cosmetics industry, the rising demand for natural and sustainable products has encouraged the incorporation of *C. ternatea* extracts into shampoos, soaps, creams, and makeup formulations. This leverages their antioxidant and soothing properties as well as the vibrant blue coloration of anthocyanins, enhancing both the visual and functional appeal of these products. Moreover, these compounds may help protect the skin and hair from oxidative damage, supporting the development of functional cosmetics with combined sensory and therapeutic benefits [74]. Finally, the traditional use of *C. ternatea* as a natural dye for textiles and handmade paper has been revitalized in sustainable and artisanal production initiatives, emphasizing circular economy practices, floral waste valorization, and the replacement of synthetic dyes in the textile and craft sectors [1].

## 5. Conclusions

Based on the reviewed scientific literature, *C. ternatea* is a versatile plant with emerging and sustainable applications across pharmaceutical, cosmetic, sensory, and artisanal sectors, positioning it as a high-value natural resource. Extracts from various parts of the plant have demonstrated multiple health-promoting effects, including antioxidant, antidiabetic, anti-obesity, hepatoprotective, and anticancer activities. They also have applications as natural colorants, functional ingredients, components of active packaging, and in nutraceutical and cosmetic formulations.

Despite this potential, several challenges remain, including the scarcity of standardized protocols, the predominance of *in vitro* studies, and the limited number of *in vivo* and clinical investigations. The technological functionality of the extracts still requires validation, and regulatory approval is necessary for their use. Future research should address toxicity profiles, bioavailability, and clinical validation, as well as optimize extraction methods and formulation strategies to maximize functional properties. Practical applications include their potential use in the pharmaceutical industry for developing bioactive compounds aimed at disease prevention or therapeutic support, in cosmetics for creating natural formulations with antioxidant and coloring properties, and in food technology as natural colorants and functional ingredients in innovative products and active packaging.

## Figures and Tables

**Table 1:** Bioactive effects and food applications of *C. ternatea* in health promotion.

Study/Extraction Method	Main Results	Reference
**Antidiabetic Studies**		
Methanolic extract of *C. ternatea* petals (1:10 *w*/*v* leaf: solvent) obtained by maceration for 24 h.	The methanolic extract (3865.6 mg GAE/g) inhibited 24.7% of α-glucosidase activity at 100 μg/mL, suggesting a potential antidiabetic effect of *C. ternatea* petals.	[30]
Aqueous extract (1:25, *w*/*v*) obtained by microwave-assisted extraction of fresh petals.	At 100 μg/mL, the inhibition rates were as follows: α-amylase: 77.69% (CT-Co_3_O_4_ NPs) and 71% (CT-Au NPs) α-glucosidase: 80.35% (CT extract), 73.27% (CT-Au NPs), and 80.04% (CT-Co_3_O_4_ NPs) Xanthine oxidase: 80.05% (CT extract), 92.33% (CT-Au NPs), and 95.64% (CT-Co_3_O_4_ NPs)	[31]
Ethanolic extract prepared by maceration of *C. ternatea* flowers.	Administration of the extract resulted in significant reductions in body weight, obesity index, and Lee index, demonstrating promising effects in controlling obesity in rats.	[32]
**Anticarcinogenic Studies**		
Ethanolic extract of leaves prepared by Soxhlet extraction.	The ethanolic extract (17.7 mg GAE/g) exhibited potent cytotoxic activity against cancer cells, as indicated by low IC_50_ values. Treatment also increased mRNA levels of GAX and DIABLO while reducing NAIP1 expression, supporting the involvement of pro-apoptotic pathways in the extract’s action.	[33]
Extract of *C. ternatea* obtained by maceration in ethanol.	The extract exhibited high cytotoxic activity against the T47D breast cancer cell line (IC_50_ = 5.21 µg/mL).	[34]
**Hepatoprotective Studies**		
Ethanolic extract of flowers obtained by maceration.	The most pronounced effects were observed with the highest extract dose (800 mg/kg). Treatment increased hepatic antioxidant enzyme activities (GSH--Px and GST) and significantly reduced alkaline phosphatase levels, indicating protection of the liver against hyperglycemia- and dyslipidemia-induced damage. Additionally, markers of kidney injury, including blood urea, serum creatinine, and uric acid, were reduced.	[35]
Ethanolic extract of flowers obtained by maceration.	Administration of the extract significantly reduced levels of aspartate aminotransferase, alanine aminotransferase, and bilirubin.	[36]
**Anti-obesity Studies**		
Aqueous extract of petals (0.125:25, *w*/*v*) obtained under stirring at 40 °C for 30 min.	The extract protected C57BL/6 mice against obesity, oxidative stress, and inflammation induced by a high-fat, high-fructose diet. It also enhanced reverse cholesterol transport by increasing HDL-C and decreasing LDL-C levels.	[37]
Aqueous extract of dried flowers (1:20, *w*/*v*) obtained by double boiling at 90–95 °C for 4 h.	Acute consumption of a high-fat meal with extract reduced postprandial serum triglycerides and free fatty acids. The extract significantly enhanced plasma antioxidant status, increasing FRAP, thiol levels, and endogenous antioxidant enzyme activity, including glutathione peroxidase. However, it did not attenuate postprandial hyperglycemia or the rise in pro-inflammatory cytokines.	[38]
Aqueous flower extract obtained by heating at 60 °C and incorporated into the herbal beverage.	An *in vivo* study in obese mice showed reduced body weight and improved lipid profile.	[39]
**Antioxidant Studies**		
Aqueous flower extract obtained by maceration for 2 h.	The extract exhibited high antioxidant activity and potential protective effects against bisphenol A-induced oxidative damage on reproductive performance, improving pregnancy rates and litter size.	[40]
Aqueous extract of dried flowers (1:20, *w*/*v*) obtained by double boiling at 90–95 °C for 4 h.	Significant increases in plasma antioxidant capacity (plasma iron reducing capacity (FRAP), oxygen radical absorbance capacity (ORAC), trolox equivalent antioxidant capacity (TEAC), and protein thiol) and decreases in malondialdehyde (MDA) levels were observed in individuals receiving 1 g and 2 g of *C. ternatea* flower extract (CTE). Furthermore, CTE consumption protected sucrose-induced reductions in ORAC, TEAC, and MDA.	[41]
**Anti-inflammatory Studies**		
Ethanol extract of the roots obtained using a Soxhlet extractor.	The ethanolic extract of *C. ternatea* (EECT) demonstrated a significant reduction in the mean paw edema volume in both carrageenan- and histamine-induced inflammation. A considerable decrease in paw diameter was observed in the EECT (200 and 400 mg/kg) and diclofenac (10 mg/kg) treated groups after day 7. Diclofenac (10 mg/kg) and EECT (400 mg/kg) demonstrated a significant reduction in paw diameter from day 14 compared with the CFA control (*p* < 0.001).	[42]
Ethanolic flower extract obtained by ultrasonic extraction at 60% power for 15 min.	The extract demonstrated anti-inflammatory, antimicrobial, and antioxidant activity in silico. The anthocyanin compounds in *C. ternatea* have anti-inflammatory effects by inhibiting or reducing the activity of pro-inflammatory proteins like TNF-α, NFκB, RANKL-RANK, and IL-6, which can trigger the upregulation of anti-inflammatory proteins such as IL-10.	[43]
Methanolic extract of fresh flowers prepared for 3 h.	The results showed significant inhibition of the COX-1 and COX-2 enzymes, with values of 82.74 ± 1.42% and 85.29 ± 1.67%, respectively, at a concentration of 200 µg/mL.	[44]

**Table 2:** Reported biological effects of *C. ternatea* and the pathways involved.

Effect	Type of Study	Compounds Involved	Pathways/Mechanisms	Reference
Anticancer	*In vitro*	Anthocyanins	Apoptosis induction, ROS modulation, NF-κB inhibition	[65]
Anticancer	*In vitro* and *In vivo*	Flavonoids, anthocyanins	Suppression of fatty acid synthesis via SREBP1 pathway, enhancement of cisplatin efficacy	[66]
Anti-obesity	*In vivo*	Anthocyanins	Modulation of lipid metabolism, activation of AMPK, and reduction in oxidative stress	[37]
Anti-inflammatory	*In vitro*	Ternatin Anthocyanins	Inhibition of NF-κB nuclear translocation, reduction in iNOS expression, and NO production	[67]

## Data Availability

No new data were created or analyzed in this study.
